# MRI-Guided Focused Ultrasound for the Treatment of Dystonia: A Narrative Review

**DOI:** 10.7759/cureus.54284

**Published:** 2024-02-16

**Authors:** Sheikh Muktadir Bin Momin, Kristian Aquilina, Harry Bulstrode, Takaomi Taira, Suneil Kalia, Ammar Natalwala

**Affiliations:** 1 Institute of Inflammation & Ageing, University of Birmingham, Birmingham, GBR; 2 Department of Neurosurgery, Queen Elizabeth Hospital, Birmingham, GBR; 3 Department of Paediatric Neurosurgery, Great Ormond Street Hospital, London, GBR; 4 Department of Neurosurgery, Wellcome-MRC Cambridge Stem Cell Institute, Addenbrooke’s Hospital, Cambridge, GBR; 5 Department of Neurosurgery, Tokyo Women's Medical University, Tokyo, JPN; 6 Division of Neurosurgery, Toronto Western Hospital, University Health Network, University of Toronto, Toronto, CAN; 7 Victor Horsley Department of Neurosurgery, National Hospital for Neurology and Neurosurgery, London, GBR; 8 Department of Neuromuscular Diseases, Institute of Neurology, University College London, London, GBR

**Keywords:** functional neurosurgery, focused ultrasound, movement disorders, parkinson's disease, dystonia

## Abstract

Contemporary surgical management of dystonia includes neuromodulation via deep brain stimulation (DBS) or ablative techniques such as radiofrequency (RF) ablation. MRI-guided focused ultrasound (MRgFUS) is an emerging modality that uses high-intensity ultrasound to precisely ablate targets in the brain; this is incisionless, potentially avoiding the surgical risks of a burr hole and transcortical tract to reach the anatomical target. There is some evidence of efficacy in essential tremor and Parkinson’s disease (PD), but, to date, there is no study aggregating the evidence of MRgFUS in dystonia. In this narrative review, we searched Medline, Embase, CINAHL, EBSCO, and ClinicalTrials.gov for primary studies and clinical trials on MRgFUS in the treatment of dystonia. Data were analyzed concerning dystonia phenotype, reported outcomes, and complications. PD-related dystonia was also included within the scope of the review. Using our search criteria, six articles on the use of MRgFUS in adult dystonia and three articles on the use of FUS in dystonia in PD were included. Four trials on the use of FUS in dystonia were also found on ClinicalTrials.gov, one of which was completed in December 2013. All included studies showed evidence of symptomatic improvement, mostly in focal hand dystonia; improvements were also found in dystonia-associated tremor, cervicobrachial dystonia, and dystonia-associated chronic neuropathic pain as well as PD-related dystonia. Reported complications included transient neurological deficits and persistent arm pain in one study. However, the evidence is limited to level-4 case series at present. MRgFUS is an emerging modality that appears to be safe and effective, particularly in focal hand dystonia, without major adverse effects. However, the quality of evidence is low at present, and long-term outcomes are unknown. High-quality prospective studies comparing MRgFUS to other surgical techniques will be useful in determining its role in the management of dystonia.

## Introduction and background

Dystonias are characterized by involuntary and disorganized movement that results from sustained or intermittent muscle contractions [1]. Dystonia can be isolated or combined with other neurological disorders [2] and, depending on the extent of the affected body regions, subclassified into focal, segmental, or generalized dystonia. Most isolated dystonias in adults are idiopathic, and a proportion of adults are known to have the associated genetic mutations [1]. The clinical phenotype of dystonia patients can vary markedly. For example, in DYT-TOR1A dystonia, an autosomal dominant hereditary condition, there is a penetrance of 30% following a heterozygous mutation in the TOR1A gene. This mutation can lead to either a variable degree of early onset, progressive, generalized torsional dystonia, or no motor phenotype in asymptomatic carriers. This variability extends to non-motor symptoms of dystonias, including pain, sleep, and psychiatric and cognitive symptoms [3]. Phenotypic variability makes it challenging to create one set of unifying pathophysiological or diagnostic criteria [4], and the diagnostic difficulty contributes to the fact that the true prevalence of dystonia is unknown. The reported prevalence of all primary dystonias is 16.43-30.9 per 100,000 [5-7], with the prevalence for dystonia subtypes ranging from 0.40 (laryngeal dystonia) to 30.9 (cervical dystonia) per 100,000 [7].

Classically, the underlying pathophysiology of dystonia has been considered to occur secondary to basal ganglia dysfunction, such as other movement disorders, centered on reports of dystonic syndromes arising from lesions involving the basal ganglia [8]. However, recent theories purport a “circuitopathy” affecting multiple anatomical regions connected by the basal ganglia-cerebello-thalamo-cortical circuits, based on the observation that functional neuroimaging studies do not demonstrate abnormalities in discrete brain regions in dystonia patients [1,9,10]. Interestingly, a recent preclinical study by the Brownstone group showed that spinal circuit dysfunction alone was sufficient to cause dystonic-like features in a Dyt-Tor1a mouse model [10].

Nonetheless, in current practice, deep brain stimulation (DBS) of the globus pallidus internus (GPi) is an established surgical treatment option for dystonia patients [11-13]. Although less common, DBS has also been used in pediatric primary and acquired dystonias (including cerebral palsy), with observed improvements in the Burke-Fahn-Marsden Dystonia Rating Scale (BFMDRS) scores [14]. Stereotactic lesional surgery, including radiofrequency (RF) ablation, is an alternative surgical technique predating DBS; since the 1950s, targets in the thalamus (including the ventro-oral (VO), ventralis intermedius (ViM), and ventral posterolateralis (VPl) regions) and the GPi have been reported [15,16]; lesional surgery was largely superseded by DBS in contemporary practice because of irreversibility of the lesions and inability to adjust stimulation parameters. Moreover, bilateral pallidotomy has associated side effects including dysarthria, dysphagia, hypophonia, and a risk of symptom recurrence [15,17]. RF ablation may still have a role in patients contraindicated for DBS [18]. Both DBS and RF ablation require a skin incision and the insertion probe or electrode into the target. DBS also requires lifelong follow-up and hardware maintenance. Dystonia patients with DBS have an increased rate of lead fracture or failure [19], although this may not be a widespread phenomenon.

FUS is a technology that harnesses ultrasound to deliver multiple focused beams of energy accurately and precisely at a specified target in the body to create a thermal lesion [20]. This technique is also referred to as “high-intensity frequency ultrasound” (HIFU). The use of ultrasound is ubiquitous in medicine, with FUS being used experimentally since the 1930s, progressing to FDA approval of the Insightec Exablate 2000 HIFU system to treat uterine fibroids in 2004 and essential tremor (ET) in 2016 [21,22]. Targeted tissue can be heated beyond 60 degrees Celsius, and the high-intensity beam can target tissue to a volume of approximately 1 mm in depth and 10 mm in length, sparing surrounding tissue [20,23]. The therapeutic mechanisms of action include thermo-coagulative necrosis and cavitation. The latter is a mechanical phenomenon where ultrasound waves generate pressure variations in fluids to create and collapse bubbles within the tissue, which can cause high temperatures, high shear stresses, and microscopic streaming, leading to apoptosis [24]. FUS may also confer a neuromodulation effect on its targeted tissue, with the Piezo1 gene implicated in this mechanism in a murine model [25].

In contemporary practice, the frequency of ultrasound can be adjusted to the depth of tissue, with lower-frequency waves allowing greater penetration into deeper tissue; trans-cranial applications use frequencies as low as 0.5 MHz, while up to 8 MHz has been used for prostate applications. Concurrent magnetic resonance imaging (MRI) may be used to ensure precise stereotactic targeting of the site of interest, termed magnetic resonance imaging-guided FUS (MRgFUS). During the delivery of MRgFUS, thermometry can be employed to measure the incremental increase in the temperature, providing a real-time temporospatial measurement (1 degree Celsius/1 mm/1 second resolution) of thermal energy at the target [20].

There is increasing evidence for the successful use of MRgFUS through targeting traditional surgical anatomical sites in adults, including targeting the ventralis intermedius (ViM) nucleus for ET and targeting the posterior thalamic central lateral nucleus in chronic neuropathic pain [26,27]. Increased interest in ablating traditional targets has also expanded to Parkinson’s Disease (PD) with unilateral subthalamic nucleus (STN)[28] and GPi [29], although this is still not widely used. Bilateral pallidothalamic tractotomy (PTT) has also been performed using MRgFUS in PD [30]. There is a paucity of evidence on the use of MRgFUS in the pediatric population at present [31], and there are few published studies on the use of MRgFUS in dystonia. In this narrative review, we will outline the current evidence on using MRgFUS to treat dystonia and discuss future possible directions for MRgFUS in movement disorders.

## Review

Materials and methods

The use of MRgFUS has gained significant traction both in the research and clinical settings. A PubMed search for the term “high intensity focused ultrasound” revealed that out of the 4543 articles in the database, 3,225 have been published since 2012. To evaluate the current evidence on the use of FUS in dystonia, a comprehensive literature search was conducted in the main medical journals and trial databases (Medline, EMBASE, CINAHL, EBSCO, and ClinicalTrials.gov) with the following terms: “Focused ultrasound” AND “dystonia”.

This exploratory search yielded 30 articles and, therefore, we included all studies describing the use of FUS in dystonia, published at any time. Three review articles were published in Japanese and were excluded. Reference lists were also inspected for inclusion.

Current Evidence for FUS in Dystonia

Using our search criteria, six articles on the use of FUS in dystonia and three articles on the use of FUS in dystonia in PD were included. Four trials on the use of FUS in dystonia were also found on ClinicalTrials.gov, one of which was completed in December 2013, and the others were either recruiting or ongoing. These studies have been summarized in Tables 1-3. The current evidence on dystonia is limited to level-4 case series and reports in 18 patients. The oldest study was a clinico-investigative study conducted by Fry et al. in 1958, in an era before CT and MRI [32]. Remarkably, they used a focused four-beam ultrasound device adapted from animal studies, using anatomical landmarks demonstrated by radio-opaque ventriculography. Their technique involved using a local anesthetic to raise a bone flap and apply the ultrasound probe directly onto the dura. In their series of 18 patients with PD and two patients with cerebral palsy-associated dystonia, they found that targeting discrete anatomical regions resulted in reduced PD symptoms and athetotic movements in cerebral palsy-associated dystonia, although the anatomical target in the dystonia patients was not specified.

**Table 1:** Articles on focused ultrasound in dystonia ADDS: Arm dystonia severity scale; CRST: Clinical Rating Scale for Tremor; PD: Parkinson’s Disease; TMDS: Tubiana Musician’s Dystonia scale; VO: Ventro-oral; WCRS: Writer’s Cramp Rating Scale

Reference	Patients (total in study)	Dystonia phenotype	Anatomical target	Outcome measures	Results	Additional comments
Horisawa et al., 2021 [33]	10 (10)	Focal hand dystonia (severe) - musician’s dystonia, writer’s cramp, professional dart-related	VO thalamus	1. Writer’s cramp rating scale (WCRS), 2. Tubiana Musician’s Dystonia scale (TMDS), 3. Arm dystonia severity scale (ADDS)	WCRS, TMDS, and ADDS all improved from 6.3 (± 2.7), 1.4 (± 0.5, and 58.7% 14.3% at baseline to 1.6 ± 3.1 (p=0.011), 5.0 ± 0 (p=0.0001), and 81.6% ± 22.9% (p=0.0229) at 12 months, respectively	Dysarthria: three transient, one at 12 months (mild) Facial palsy - two transient
Horisawa et al., 2018 [34]	1 (1)	Musician’s dystonia	VO thalamus	TMDS	Improvement in TMDS from 1 (/5) to 4 at one week, 5 at three months	No complications
Meng et al., 2018 [39]	1 (1)	Task-specific dystonia (writer’s cramp)	VO and ViM	Clinical Rating Scale for Tremor (CRST)	Improvement from 30 (/152) at baseline to 1, 2, and 2 at one month, three months, and six months, respectively	Dysesthesia to the right side of the tongue (persistent) at six months, clumsiness/gait disturbance (resolved)
Fasano et al., 2017 [38]	3 (6)	Cervicobrachial dystonia, writer’s cramp, and dystonia gene-associated tremor (3 tremor-dominant PD)	ViM thalamus	Tremor rating scale (TRS), DRS	Improvement in contralateral TRS-A and TRS-B was 17 (±2.7) at baseline, reduced by 42.2% at one week and 52.9% at six months (p<0.05)	Transient dizziness, numbness, poor balance, and nausea were reported as side effects. The writer’s cramp patient had persistent hemi-tongue numbness (presumed contralateral) at six months. One PD patient died of pneumonia four months after surgery.
Martin et al., 2009 [27]	1 (9)	Neuropathic pain from cervical dystonia	Central lateral thalamus	Clinical assessment	Mean pain improvement by 68% two days post-procedure	“Vestibular feelings”, paresthesia, dysesthesia, and somatosensory improvements were reported at 48-hour follow-up.
Fry et al., 1958 [32]	2 (20)	Cerebral palsy-related dystonia	The whole cohort: ansa lenticularis, the medial segment of globus pallidus; substantia nigra; medial STN; tegmental fields of Forel	Clinical assessment	Reduced athetotic movements and fluctuating muscle tension without impairing voluntary movement	No intraoperative mortality; changes in deep and superficial reflexes, vibratory perception, and touch perception; changes in motor power, coordination and posture, and rigidity; changes in vital signs, responsiveness

**Table 2:** Articles on dystonia in Parkinson’s disease MDSP: Movement Disorder Society of the Philippines; PD: Parkinson’s disease; XDP: X-linked parkinsonism

Reference	Patients (total in study)	PD-dystonia phenotype	Anatomical target	Outcome measures	Results	Additional notes
Gallay et al., 2021 [30]	5 (10)	Treatment-resistant PD	Pallidothalamic tract (bilateral)	Clinical assessment	Dystonia suppressed in four out of five patients at one year	One patient had hiccups up to ten months after therapy, and one had gait disturbance immediately after the procedure, lasting until three months. One patient had episodes of uncontrollable laughter and blepharospasm at one year
Jamora et al., 2021 [50]	3 (3)	X-linked dystonia-parkinsonism	Pallidothalamic tract (unilateral)	XDP-Movement Disorder Society of the Philippines (MDSP) Scale	Improvement in XDP-MDSP dystonia scale from mean 18.7 to 15 at six months (19.7%), overall improvement of 30.1% at seven months	Two cases had persistent right arm pain, requiring tramadol (at two and seven months)
Chang et al., 2019 [51]	1 (1)	X-linked dystonia-parkinsonism	Pallidothalamic tract (unilateral)	Clinical assessment	No neurological deficits after treatment	No reported adverse effects

**Table 3:** Registered clinical trials on FUS in dystonia BFMDRS: Burke-Fahn-Marsden Dystonia Rating Scale; GPi: globus pallidus internus; MDSP: Movement Disorder Society of the Philippines; TWSTRS: Toronto Western Spasmodic Torticollis Rating Scale; UPDRS: Unified Parkinson’s Disease Rating Scale; XDP: X-linked Parkinsonism

Reference	Planned number of patients	Dystonia phenotype	Anatomical target	Outcome measures	Results	Current status
Jamora, 2022 [41]	Undefined	X-linked parkinsonism dystonia	Pallidothalamic tract	XDP-MDSP scale, pre- and post-treatment UPDRS, BFMDRS	Pending	Recruiting
Lozano, 2014 [42]	10	All treatment-refractory movement disorders (dystonia, PD, ET)	Undefined	Primary: severity of device- and procedure-related complications	Three published studies: two on ET, one on non-ET tremor syndromes	Ongoing
Martin et al., 2012 [44]	10	All treatment-refractory movement disorders (dystonia, PD, ET)	Medial thalamic nuclei, STN, globus pallidus	Primary: lesion size and patient safety. Secondary - clinical efficacy and QoL	Unknown	Completed December 2013
Horisawa, 2021 [43]	10	Cervical dystonia	Pallidothalamic tractotomy	Primary: Toronto Western Spasmodic Torticollis Rating Scale (TWSTRS) at six months	Pending	Ongoing

Contemporary articles have largely centered on the treatment of focal hand dystonia (FHD), a sporadic focal dystonia that usually presents in patients between 30 and 50 years of age, with clinical phenotypes ranging from writer’s cramp to dystonia [2]. Approximately 13 out of 16 patients from the five recent studies were treated for FHD, with 11 patients undergoing MRgFUS to the Vo thalamus [33,34], a previously established anatomical target for ablative procedures for FHD [35-37]. Additionally, two patients with tremor-dominant writer’s cramp underwent ViM thalamotomy (one ViM only and one combined ViM/VO thalamus) [38,39], as ViM is an established anatomical target for tremor [40]. Of the three remaining patients, two patients underwent ViM thalamotomy for cervicobrachial dystonia and tremor associated with a dystonia gene, which was unspecified, respectively [38], and one patient had central lateral thalamotomy to treat a neuropathic pain syndrome associated with cervical dystonia [27]. In three articles, patients with and without dystonia were included, reporting outcomes with respect to the whole group [27,32,38]. Table 1 provides a summary of the clinical outcomes for these studies, which all showed a symptomatic improvement following FUS.

All contemporary articles described performing MRgFUS under local anesthetic in a 3-Tesla MRI machine (Magnetom Verio, Siemens Healthcare) using the Insightec ExAblate FUS platform (Insightec Ltd., Tirat Carmel, Israel). All contemporary studies described applying sonications to the desired anatomical target at a lower temperature of approximately 40 degrees Celsius with subsequent increases in temperatures until therapeutic effects were reached or side effects observed. Patients were monitored clinically after each temperature increase, and temperature changes at the anatomical target were tracked with MRI thermometry. There are currently four registered clinical trials on MRgFUS on ClinicalTrials.gov (Table 3). Of these, three are ongoing; two are conducted by research groups who have previously published studies related to X-linked parkinsonism-dystonia (XDP) and non-ET tremor syndromes [41,42], while another is investigating MRgFUS PTT for cervical dystonia [43]. The completed trial [44] does not currently have any published outcomes.

Evidence of Efficacy in Treating Dystonia

Most recent studies on MRgFUS in dystonia have focused on three different outcome measure scales; the dystonia rating scales [33], tremor rating scales [38], and clinical assessment for pain [27]. At a group level, all outcome measures showed immediate improvement following MRgFUS therapy, which was sustained in the short- and long-term follow-up period. Statistically significant improvements were reported in dystonia measures and tremor outcomes in the two case series [33,38]. All but one dystonia patient in all included studies was observed to have had an objective benefit on clinical and dystonia/ tremor rating scale assessment. One patient in the FHD case series had poor treatment outcomes. Moreover, this patient also had a history of mental health disorders prior to the treatment and attempted suicide two months after treatment [33]. Only one study reported functional outcomes, with four out of five of the FHD case series returning to work at 12-month follow up [33]. There was an appreciable qualitative and quantitative improvement in the handwriting of patients with writer’s cramp and instrument playing in other included studies [34,38,39]. Notably, in these studies, while the reported complication rate of MRgFUS is low, the number of patients undergoing this mode of treatment is also low.

Dystonia in Parkinsonism

Dystonia may be found in up to 30% of patients with PD, particularly early-onset PD [45], and dystonia symptoms are included as part of routine Unified Parkinson’s Disease Rating Scale (UPDRS) assessment [46], although PD dystonia differs phenotypically from other forms of dystonia. MRgFUS in PD is better established compared to dystonia, with the ViM nucleus, and to a lesser extent, the GPi and STN being targeted successfully [47]. The FDA approved ViM MRgFUS for tremor-dominant PD alongside ET in 2018, followed by unilateral GPi MRgFUS for dyskinesia, rigidity, or mobility symptoms in 2021 [48,49]. During our search, three studies related to dystonia in PD were found [30,50,51]. Two of the included articles in our review report patients with XDP, a rare neurodegenerative disorder found in males from Panay Island, Philippines, largely manifesting as dystonia [52]. In these studies, four patients underwent unilateral pallidothalamic tract MRgFUS using a similar operation protocol as described in dystonia studies outlined in the previous section, using a 1.5-Tesla MRI system. In the series with three patients, there was an average improvement in the total XDP-MDSP (Movement Disorder Society of the Philippines) scale by 30% at six months [50]. The dystonia subscale improved by 34.2% at one month but regressed to 19.7% improvement at six months. Although there was incomplete follow-up beyond this point, overall dystonia improvement was observed to be sustained at 12 months. All subcomponents of the score showed improvement aside from component 3B (nonbehavioral), which included patient/carer-reported outcomes on sleep, pain, bladder incontinence, fatigue, and salivation/ drooling. The performance in component 3B was influenced by two patients experiencing persistent arm pain during the follow-up period.

Another study evaluated ten patients with mixed- or tremor-dominant medication-resistant PD, five of whom had developed dystonic symptoms [30]. This group received bilateral pallidothalamic tract MRgFUS, resulting in significant reductions in UPDRS measures of mean tremor, rigidity, and distal hypo-bradykinesia (91%, p=0.006; 67%, p=0.006; 54%, p=0.01, respectively), but having a negative impact on speech, no impact on axial items, and a nonsignificant improvement in gait and postural instability. Four out of five patients with dystonia associated with their PD had dystonic symptoms suppressed following treatment and all four patients with dyskinesias. The outcomes of these studies are summarized in Table 2.

Adverse Events From MRgFUS for Dystonia

Transient and long-term side effects were reported in all studies, usually sensorimotor in origin. Among 11 patients who had VO thalamotomy, four had dysarthria (one persisted beyond 12 months), and two had transient facial palsy attributed to encroachment of the ablative lesion onto the internal capsule, which resolved in three months [33]. Both patients with writer’s cramps who had solely ViM or combined ViM/VO thalamotomy experienced persistent sensory disturbances to the hemi-tongue. Transient balance/ gait problems were reported in one patient with writer’s cramp and two tremor-associated dystonia patients [34,38]. Other transient side effects are described in Table 1.

Postprocedural Dystonia Following MRgFUS for Tremors

Dystonia is a recognized complication following thalamic neurosurgery [53]. During our search, we found two studies that reported dystonia following MRgFUS thalamotomy for tremor. In one case series of 12 patients with medically refractory ET or ET-plus syndromes who underwent MRgFUS ViM thalamotomy, two patients with upper limb and head/neck tremor (one with childhood-onset, and one presenting at 77 years of age with a 20-year history of familial tremor) with associated dystonia (right upper limb posturing and torticollis, and multifocal dystonia, respectively) were evaluated [54]. Both patients had improvement of their tremor measurements, but worsening of dystonia measurements, leading to worsening function compared to preoperatively. This phenomenon was not observed in other cases with “no” or “slight” dystonia as per the BFMDRS score.

In another case report, a 70-year-old man with a background of ET affecting his upper limbs and voice, with no baseline signs of dystonia was presented. He underwent a left MRgFUS between the ventro-posteriolateral/posteriomedial/lateral and mediodorsal thalamus, which reduced his right hand and postural tremor at a 10-month follow-up. However, at 17 months post-procedure, he developed dystonic posturing and a new low frequency (3Hz), low amplitude tremor in the right hand, and a 5Hz tremor in the left hand, which was absent at rest [55]. Although the underlying mechanism of this complication is unknown, it may be a separate deleterious effect of ablation of the thalamus, possibly targeting the cerebellothalamic and the cortico-striato-pallidothalamo-cortical loops [53]. Although one of the cases (the 77-year-old patient with familial tremor) was noted to have a similar thalamic lesion size in MRI compared to other patients, the size and precision of the lesions were not commented on in the other two cases and may also have influenced the development of these complications. Thus, careful assessment of associated dystonia should be undertaken in patients presenting with tremors for possible surgical evaluation.

Discussion

In this narrative review, we explored the current evidence for the use of MRgFUS in dystonia. As a novel technology, the evidence is limited only to six level-4 case series and reports covering a total of 18 patients. Moreover, three case series in nine patients with PD-related dystonia were also included. The current evidence suggests that MRgFUS in dystonia is safe and effective in reducing focal dystonia [33,34], tremor [38,39], and neuropathic pain [27] symptoms. Included studies largely reported on patients with FHD, although patients with dystonia-gene-associated tremor, cervicobrachial dystonia, and PD-related dystonia were also evaluated [30,50,51]. Studies on FHD mainly treated dystonias in distal limbs rather than axial dystonias (e.g., cervical, limb-girdle, or generalized). Included studies in PD-related dystonia targeted the pallidothalamic tract with good efficacy, with this being the target of an ongoing clinical trial (Tables 2-3) [30,41,50,51].

FUS exerts a thermo-coagulative and cavitation effect to cause permanent ablation, akin to RF ablation. RF pallidotomy and thalamotomy are known treatments for dystonia, but when applied bilaterally, this technique can be associated with dysarthria in up to 30% of patients; therefore, unilateral ablation is often recommended [56]. Most of the included MRgFUS articles described unilateral ablation, with one article on MRgFUS PTT thalamotomy in XDP highlighting this as an inherent limitation of the technique [50]. However, another series described staged bilateral PTT thalamotomy in 10 patients with treatment-resistant PD who had responded successfully to prior unilateral thalamotomy, with contemporaneous bilateral surgery undertaken in select patients considered to have intact “thalamocortical reserves,” defined as an absence of brain atrophy and normal cognitive status [30]. Although there was a reported reduction in UPDRS-III, there was no significant change in measures of gait and an increase in speech disturbance (hypophonia, tachyphemia, and speech initiation), as shown in Table 2. Interestingly, only one patient was noted to have speech disturbance at two days post-second surgery, increasing to five patients with objective speech disturbance at one year. Hence, bilateral MRgFUS appears to possess similar risks to other forms of bilateral ablative surgery.

Akin to RF ablation, MRgFUS does not produce any radiation, can be conducted under local anesthetic, and provides a versatile alternative employing incremental temperature increases at the target site to titrate treatment to effective relief of symptoms or development of side effects [57]. Compared to DBS or RF ablation, MRgFUS may be a suitable option in frail, older populations, but current data are limited [58]. Moreover, alongside this ablative mechanism, FUS may have a neuromodulation mechanism, with the Piezo1 gene implicated [25].

There are several other important factors to consider in MRgFUS. Firstly, as an ablative method, it causes permanent change to the target anatomical area. Histology obtained from a patient with tremor-dominant PD who died from a fall 10 days after successful MRgFUS thalamotomy demonstrated central necrosis and cytotoxic oedema at the center with surrounding vasogenic oedema, correlating to MRI-thermography findings [59]. A small proportion of patients may have significant persisting gait, speech, and sensorimotor disturbances that may persist for up to 12 months in both MRgFUS treatments for ET and PD ET [26,28]. Our review demonstrated similarly low rates of adverse events, mainly transient; however, most studies have relatively short endpoints. Interestingly, two patients with writer’s cramps from separate studies (treated at the ViM and ViM/ VO thalamus) experienced unilateral tongue numbness. This may relate to the complexity of targeting the ViM thalamus, which is not readily visible in conventional MRI sequences, but target visualization may be improved using probabilistic tractography [60]. Furthermore, all studies had follow-up to a maximum of 12 months and, thus, outcomes beyond this point are unknown. Of note, a three-year follow-up study of STN-MRgFUS in PD showed sustained efficacy with no delayed or disabling adverse events [61], suggesting a possible medium-term clinical benefit.

Additionally, the differing efficacy of MRgFUS in managing specific dystonia (and/or dystonia tremor) symptoms should direct clinicians towards selecting patients and anatomical targets accordingly. VO thalamotomy was broadly successful in the treatment of FHD with an acceptable side-effect profile [62]. In comparison, ViM thalamotomy was shown to be effective in treating dystonic tremors [38,39], akin to its efficacy in treating other tremor syndromes. A meta-analysis on PD-related tremor found that ViM-MRgFUS significantly improves UPDRS-III tremor scores on and off-medications and was non-inferior to DBS in varying locations [63], suggesting the ViM may be a suitable anatomical target for tremor syndromes. Interestingly, we found no MRgFUS studies targeting the GPi for the indications, nor any comparisons to any patients who had undergone DBS or RF ablation for FHD, or other dystonia syndromes reported in the studies. A recent randomized clinical trial explored the merit of unilateral pallidal MRgFUS for PD, showing proof of concept, and a reporting improvement in motor outcomes [29]. However, motor improvement was defined using a relatively small change on the Movement Disorders Society-UPDRS (MDS-UPDRS) or Unified Dyskinesia Rating Scale post-intervention, relative to previous trials reporting larger improvements in rating scores following RF pallidotomy, in patients with more severe disease [64,65]. PTT using MRgFUS, particularly for axial dystonias, may be more favorable than the GPi as there are no critical nearby structures such as the pyramidal tract and optic pathway. Moreover, as the pallidothalamic tract is more midline than GPi (PTT: 8 mm, GPi: 20 mm), achieving a consistent temperature rise is more technically feasible [66,67].

Our review found no articles describing the use of MRgFUS in pediatric dystonia, either genetic or acquired (including cerebral palsy). In pediatric populations, the implications of a permanent lesion enacted through an ablative procedure such as MRgFUS may have different ethical and practical implications but may be considered acceptable in children with a poor neurological baseline. A recent study in three cerebral palsy patients aged 14-22 years of age undergoing cerebellar dentate nuclei deep brain stimulation demonstrated improvement in subjective and objective dystonia motor scores [68]. This may be a future potential target for MRgFUS with the theoretical advantages of avoiding the hardware maintenance and other complications existing with pediatric DBS, including revisional procedures such as battery changes or lead lengthening.

## Conclusions

In conclusion, there is little high-quality evidence on the use of MRgFUS in dystonia, although the literature provides evidence of its safety and efficacy. Patient selection for treatment with MRgFUS is as important, as it is in other functional neurosurgical procedures, and, in the modern era, should also incorporate a molecular and clinical classification of dystonia to standardize patient selection, the anatomical target, and MRgFUS treatment protocol and follow-up for comparable results. Prospective studies on larger cohorts of patients with dystonia undergoing MRgFUS and comparative studies with other surgical modalities (e.g., DBS, RF ablation) are indicated to elucidate the role of MRgFUS in the management of these conditions, as well as consideration of MRgFUS as a “rescue” procedure in severe dystonia.
